# A Chondroitin Sulfate–Iron Complex with Antibacterial Activity and Its Derived Hydrogel for Infected Wound Healing

**DOI:** 10.3390/gels12040329

**Published:** 2026-04-15

**Authors:** Qingshan Shen, Yujie Dong, Jiawen Li, Jiarui Wu, Chengzhi Hu, Yang Liu, Lei Zhao, Huan Zhan, Hua Bian, Yanli Ma

**Affiliations:** 1 Henan Key Laboratory of Zhang Zhongjing Formulae and Herbs for Immunoregulation, Zhang Zhongjing College of Chinese Medicine, Nanyang Institute of Technology, Changjiang Road 80, Nanyang 473004, China; shenqs973@163.com (Q.S.); 17623418419@163.com (Y.D.); 17303878281@163.com (J.L.); 18290350127@163.com (J.W.); liuyang3212605@163.com (Y.L.); zhaoleibvrc@163.com (L.Z.); huanzhan@nyist.edu.cn (H.Z.); biancrown@163.com (H.B.); 2 College of Food Science and Technology, Hebei Agricultural University, Baoding 071000, China; 17631242467@163.com

**Keywords:** polysaccharide–metal complex, antibacterial activity, hydrogel, wound healing

## Abstract

In this study, a hydrogel was developed based on a chondroitin sulfate–iron complex (CSFe) incorporated into a sodium alginate matrix. The CSFe complex was first prepared through the interaction of chondroitin sulfate (CS) with Fe^3+^ ions, achieving an iron content of 2.06%. Structural characterization confirmed that Fe^3+^ coordinated with the carboxyl, sulfate, and N-acetyl groups of CS, resulting in increased molecular weight and altered physicochemical properties. The CSFe complex exhibited significant antibacterial activity against *Escherichia coli* and *Staphylococcus aureus* (*S. aureus*), and was further incorporated into a sodium alginate matrix to form an injectable hydrogel with favorable physicochemical properties such as spreadability, shear-thinning behavior, and a compact porous microstructure. In a mouse model of *S. aureus*-infected wounds, the CSFe hydrogel significantly accelerated wound closure, reduced the levels of pro-inflammatory cytokines (TNF-α and IL-6), and increased the anti-inflammatory cytokine IL-10, indicating potent anti-infective and immunomodulatory functions. Overall, this work presents a multifunctional CSFe-incorporated hydrogel system that integrates antibacterial, anti-inflammatory, and tissue-regenerative properties, offering a promising strategy for infected wound healing and highlighting the potential of trivalent iron–polysaccharide coordination complexes in the development of advanced biomedical materials.

## 1. Introduction

Skin wound infections caused by *Staphylococcus aureus* (*S. aureus*) pose a considerable burden, with profound psychological and socioeconomic implications that severely compromise the quality of life for affected individuals [1,2]. *S. aureus*, the most commonly bacteria in wound contamination, can produce a biofilm that is recognized to relate to pathogenicity and drug resistance. A biofilm can protect bacteria from antibacterial substances and reduce drug penetration [3]. Moreover, bacterial infection is capable of prolonging the wound healing time and leading to chronic and non-healing wounds, and even immune signaling and inflammatory response can be directly triggered during infection [4]. How to achieve the effective treatment of infected wounds without inducing drug resistance remains a significant challenge.

Hydrogels exhibit appealing advantages in wound dressing applications, owing to their superior biochemical characteristics [5]. For instance, the strong water-retention capacity of hydrogels creates a moist wound environment that facilitates granulation tissue formation, thereby accelerating wound healing. Some recently published articles are presented in Table 1 including the hydrogels in the composition, the target and the main results. Chondroitin sulfate (CS), a sulfated glycosaminoglycan and complex linear anionic polysaccharide, is polymerized by repeating disaccharide units of D-glucuronide and N-acetylamino galactose, with abundant carboxyl and sulfate groups [6]. Generally, CS needs other crosslinking agents such as aldehyde and hydrazine [7], or to be mixed with other hydrogel materials such as sodium alginate (SA) [8] to form hydrogel. However, the lack of antibacterial activity of CS and SA restricts their use for treating wound infections. Recently, metal ion-mediated cell death [9], particularly ferroptosis, has gained significant attention as a novel antibacterial strategy [10]. A previous study indicated that CS can interact with metal ions and calcium, forming a chondroitin sulfate calcium metal complex [11]. Interestingly, metal ions, such as Ca^2+^, Mg^2+^, Mn^2+^ and Zn^2+^, can interact with CS and enhance its bioactivity [12]. We hypothesized that introducing Fe^3+^ into CS would form a chondroitin sulfate iron complex (CSFe), thereby conferring antibacterial properties to CS. Subsequently, CSFe and SA were fabricated into a hydrogel that exhibits enhanced dual antibacterial and anti-inflammatory activities for the management of infected wounds.

To address this challenge, we developed a CSFe complex-based hydrogel system with distinctive features that differentiate it from previously reported polysaccharide-metal wound dressings. Unlike most studies that focus on divalent metal ions or simple physical mixtures, we systematically characterized the coordination between Fe^3+^ and CS, identifying the specific binding sites (carboxyl, sulfate, and N-acetyl groups) and their effects on physicochemical properties. Furthermore, we further explored its antibacterial activity against *S. aureus* and *E. coli*, providing a novel antibacterial strategy distinct from conventional antimicrobial approaches. To translate this material into a clinically applicable format, we incorporated CSFe into a sodium alginate-based injectable hydrogel and evaluated its physicochemical properties, including its injectability, spreadability, and porous microstructure. Finally, we assessed the in vivo performance of this CSFe hydrogel in a mouse model of *S. aureus*-infected wounds, demonstrating its dual capacity to control infection and modulate inflammation. This work thus presents a multifunctional CSFe-based hydrogel system that integrates antibacterial, anti-inflammatory, and tissue-regenerative properties, offering a promising alternative to existing wound dressings and highlighting the potential of trivalent iron–polysaccharide coordination complexes in advanced biomedical applications.

## 2. Results and Discussion

### 2.1. Fabrication and Characteristics of CSFe

**Figure 1:**
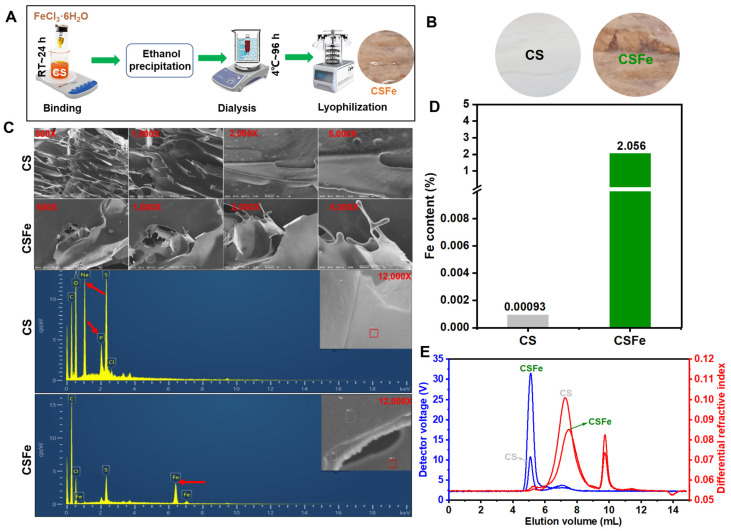
Fabrication and characteristics of CSFe. (**A**) The flow chart of the preparation of CSFe; (**B**) CSFe and CS powder; (**C**) SEM images and element mapping of CSFe and CS. The red arrows represent the signal of disappearing (Na and P) or newly appearing (Fe) elements; (**D**) Fe content (%) in CSFe and CS; (**E**) The signal chromatogram profiles of CSFe and CS from a multi-angle laser light scattering detector (blue) and the refractive index detector (red) to determine the Mw.

As illustrated in Figure 1A, the fabrication of CSFe involved four steps: the binding reaction between CS and Fe^3+^, ethanol precipitation, dialysis, and lyophilization. Afterwards, CSFe powder was obtained. Compared with the CS sample, CSFe was yellowish-brown (Figure 1B), which was attributed to the introduction of iron ions. The morphologies of CSFe and CS were similar at the same magnification, but element mapping of CSFe and CS was significantly changed (Figure 1C). Especially, the elements of Na and P from CS sample were not observed while the Fe element was observed in the CSFe sample. The Fe content detected by ICP-MS in CSFe sample was 2.06%, which was higher than that in the CS sample (Figure 1D). The Mw of CSFe was 1.48 × 10^5^ Da, which was different from that of CS (4.16 × 10^4^ Da); the chromatograms of CSFe and CS were as shown in Figure 1E. CS, a kind of anionic polysaccharide, has lots of carboxyl and sulfate groups. Normally, CS is combined with Na^+^ and K^+^ and other divalent metal ions, such as Ca^2+^, Mg^2+^, Mn^2+^, Cu^2+^, Zn^2+^, and Sr^2+^, and can interact with the carboxyl or sulfate groups to form a polysaccharide–metal complex [6]. The binding effects were attributed to the electrostatic interaction, charge density, ionic strength, and other factors. For instance, Ca^2+^ can interact with carboxyl and sulfate groups of CS, forming the potential interaction model of –OSO_3_– … Ca^2+^ … –OOC– or –COO– … Ca^2+^ … –OOC–. However, to the best of our knowledge, the research regarding interactions between CS and Fe^3+^ is limited. The results regarding the fabrication and characteristics of CSFe showed that the introduction of iron ions can change the color of CS to yellowish-brown and increase the Mw. This is probably due to the interaction between Fe^3+^ and chains of CS, causing aggregation between CS molecules.

### 2.2. Structure and Thermal Stability of CSFe

**Figure 2:**
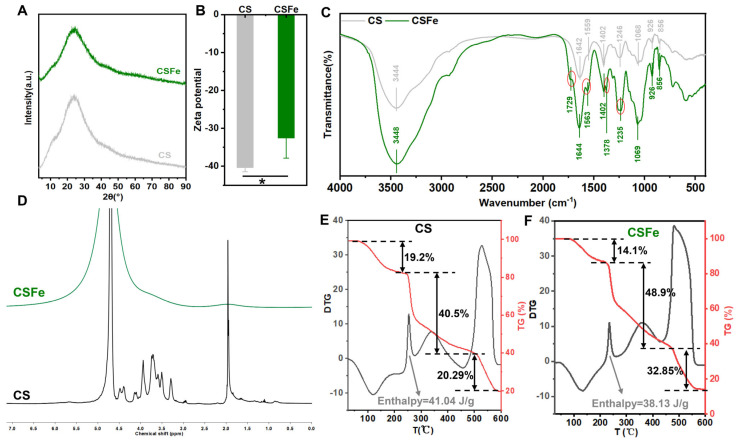
Structure and thermal stability of CSFe. XRD spectra (**A**) and Zeta potential (**B**) of CS and CSFe; FTIR (**C**); 1H−NMR (**D**) spectra of CSFe and CS; the DTG and TG curves of CS (**E**) and CSFe (**F**). The asterisk (*) indicates significant difference (*p* < 0.05). The oval borders in FITR indicate the significant changes in spectral bands (CS vs. CSFe).

To identify whether the introduction of Fe^3+^ alters the structure of CS, an XRD of CSFe and CS was performed, and the results are shown in Figure 2A. The intensity and sharpness of the XRD peaks usually indicated the sample’s crystalline nature. However, there were no sharp peaks (only a broad peak in the range of 20–40°) observed in the XRD pattern of CSFe and CS (Figure 2A). This means that the introduction of Fe^3+^ does not change the amorphous characteristic of CS, which suggests that the structure of CSFe also has an amorphous nature. The zeta potential of CS is −40.38 ± 1.10 mV, and that of CSFe is −32.62 ± 5.26 mV (Figure 2B). CS is a kind of anionic polysaccharide exhibiting negative charge in aqueous solutions while the introduction of Fe^3+^ significantly reduces the Zeta potential of CS. Generally, smaller molecules or dispersed particles exhibit higher absolute Zeta potential values (positive or negative), which correlates with greater system stability. Conversely, lower Zeta potential values increase the likelihood of aggregation. This means that the introduction of Fe^3+^ probably causes CS aggregation, which explains why the Mw of CSFe is larger than that of CS (Figure 1E). Additionally, this further illustrates that this complex formation of CSFe is attributed to the interaction of Fe^3+^ and CS, instead of the physical mixing of CS and Fe.

In order to verify the binding site of Fe^3+^ and CS, the FTIR and 1H-NMR of CSFe and CS were compared and analyzed. According to the wavenumbers, which ranged from 4000 to 400 cm^−1^ for CS and CSFe, the two spectra are overall similar with some differences observed in the range 2000–1000 cm^−1^ (Figure 2C). Notably, a broad spectral band at nearby 3440 cm^−1^ is observed in the spectra of CS (3444 cm^−1^) and CSFe (3448 cm^−1^), which is recognized as the stretching of -OH and N-H. Based on the descriptions of Uchisawaa et al., the band of unionized carboxyl groups is absorbed at 1680–1740 cm^−1^, and the ionized carboxyl group is recognized at 1550–1620 cm^−1^ [16]. Here, the strong absorption bands of the carboxyl groups are at 1644 cm^−1^ (CSFe) and 1642 cm^−1^ (CS). However, a new absorption of CSFe appeared at 1729 cm^−1^, which may be due to Fe^3+^ acting on the carboxyl group. The peaks at 1560 cm^−1^ usually represent the N-H band, suggesting the presence of -NH-C=O [17] from N-acetylglucosamine. A weak absorption band at 1559 cm^−1^ and a strong absorption band at 1563 cm^−1^ were observed in CS and CSFe spectra, respectively. The enhanced absorption at 1563 cm^−1^ of CSFe may be caused by the introduction of Fe^3+^. It is suggested that Fe^3+^ probably acts on the N-acetyl group. Absorption at 1400 cm^−1^ is characteristic of S=O stretching [18], and the asymmetric -SO_2_ stretching absorption band of CS is thought to be 1255 cm^−1^ [11]. In the CSFe spectrum, near 1402 cm^−1^ of S=O stretching, there is a new absorption at 1378 cm^−1^, and the absorption at 1235 cm^−1^ of asymmetric -SO_2_ stretching is different from that at 1246 cm^−1^ from the CS spectrum, which probably means that Fe^3+^ influences S=O stretching. Taken together, the introduction of Fe^3+^ affects the absorption bands of carboxyl groups, N-H and S=O. This means that the binding sites of Fe^3+^ and CS appear in carboxyl groups, N-acetyl groups and sulfate groups. The 1H-NMR spectra of CSFe and CS are shown in Figure 2D. The representative chemical shift in CS 1H-NMR spectrum has the range 3–5 ppm, which is similar to our previous research [11]. However, due to the magnetic properties of Fe^3+^, the representative chemical shift in CSFe is not shown, except that of the solvent (deuterium oxide).

The thermal properties of polymers, including their thermal behavior, decomposition, and heat flow during physical or chemical transitions, can be characterized using DSC [19]. The weight reductions in CS and CSFe samples occurred at three different stages. In the first stage, 19.2% of CS initial mass is lost when the temperature reaches about 250 °C in the TG curve (Figure 2E). A total of 14.1% of that is lost, and the temperature reaches about 225 °C in the TG curve of the CSFe sample (Figure 2F). The first mass loss is mainly due to the evaporation of free water from the polysaccharides. The second stage shows a sharp mass loss for CS and CSFe at about 275 °C and 260.2 °C, respectively, corresponding to random glycosidic bond cleavage and sample decomposition. Upon heating to about 600 °C at the last stage, 20.01% and 14.25% of CS and CSFe initial mass remain. The DTG curves of CS and CSFe (Figure 2E,F) present an endothermic transition process in the range of 25–180 °C corresponding to the desorption of free waters in CS and CSFe samples. The exothermic peaks in CS and CSFe at 253.3 °C and 359.6 °C, respectively, corresponding to the enthalpy values, are 41.04 J/g and 38.13 J/g, which are ascribed to the decomposition of polysaccharide. DSC results suggest that CSFe and CS have a similar thermal stability.

### 2.3. Antibacterial Activity of CSFe

**Figure 3:**
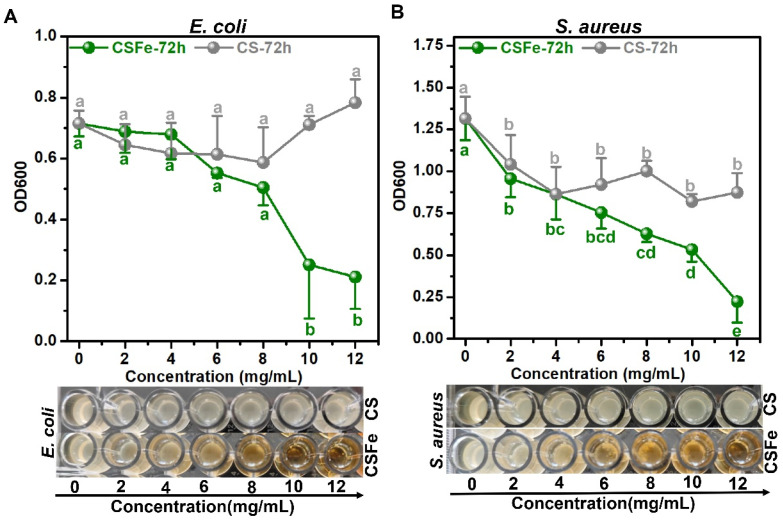
The effects of CSFe and CS on the growth of *E. coli* and *S. aureus*. The growth curve (OD600) of *E. coli* (**A**) and *S. aureus* (**B**) when exposed to CS and CSFe (0–12 mg/mL) incubated at 37 °C for 72 h. The plots below show the corresponding status of LB and TSB media containing *E. coli* (**A**) and *S. aureus* (**B**) with different concentrations of CS and CSFe. The same color and different letters indicate significant difference (*p* < 0.01).

The effects of CSFe, with CS as the control, on the growth of *E. coli* and *S. aureus* were determined by the broth microdilution method. As shown in Figure 3A, at the given concentrations (0–12 mg/mL), CS is not capable of inhibiting the growth of *E. coli*. However, CSFe, at concentration of 10 mg/mL, can significantly (*p* < 0.01) inhibit the growth of *E. coli*, and LB medium is visibly clear at this concentration. In terms of *S. aureus* (Figure 3B), although the growth curve of *S. aureus* exposed to CS (0–12 mg/mL) at OD600 shows that CS seems to inhibit the growth, the TSB medium is turbid. Nevertheless, compared with CS at the same concentration (over 6 mg/mL), CSFe is able to dramatically prohibit the growth of *S. aureus*, and the TSB medium is visible at concentrations of 10 mg/mL and 12 mg/mL. For convenience, in the following research, the MIC of CSFe to *E. coli* and *S. aureus* is defined as 10 mg/mL.

Additionally, the bactericidal activity of CSFe with *E. coli* and *S. aureus* was also assessed by the drop plate method. The MIC, 2MIC and 3MIC of CSFe, with CS as the control, were selected as the bactericidal concentrations for *E. coli* and *S. aureus*. The results are shown as Figures S1 and S2. At the given concentrations and cultured conditions (containing the normal medium), CS does not kill cells of *E. coli* (Figure S1A) and *S. aureus* (Figure S2A), and *E. coli* or *S. aureus* can grow normally. Interestingly, survival rates of *E. coli* (Figure S1B) and *S. aureus* (Figure S2B) when exposed to CSFe for 12 h at 2MIC are 6.18% and 0.03%, respectively, and the survival rates when exposed to 3MIC are 2.27% and 0.02%, respectively. This means that the bactericidal rates of CSFe at 2MIC to *E. coli* and *S. aureus* can reach 93.82% and 99.97%. Therefore, the MBC of CSFe to *E. coli* and *S. aureus* is defined as 2MIC (20 mg/mL).

The antibacterial ability of CS, especially at a low concentration, is usually very limited. For example, CS, at a concentration of 50 mg/mL, still exhibits little inhibitory activity against *E. coli* [20]. The concentrations of commercial CS reach 360 mg/mL and 420 mg/mL as the MIC, showing antibacterial activity against *E. coli* and *S. aureus* [21], respectively. Interestingly, the polysaccharide metal complex of CSFe possesses antibacterial properties against *E. coli* and *S. aureus*, with an MIC of 10 mg/mL. The inhibitory activities of CSFe, compared with that of CS, against *E. coli* and *S. aureus,* increase by 36 times and 42 times, respectively. Additionally, CSFe can indeed kill cells of *E. coli* and *S. aureus* at 2MIC, and the bactericidal rates against *E. coli* and *S. aureus* can reach 93.82% and 99.97%. It is suggested that this antibacterial ability of CSFe should be attributed to the introduction of Fe^3+^. Recently, ferroptosis has been widely studied as a hotspot. In terms of bacteria, ferroptosis-like death can occur by improving the iron levels around the bacteria, which triggers the Fenton reaction and destroys the bacterial cell membrane [22]. Based on ferroptosis, iron or ferrous ion preparations have been proposed as noise strategies to overcome antimicrobial-resistant bacterial infections such as *S. aureus* and *E. coli* [23,24,25]. Here, the antibacterial ability of CSFe against *S. aureus* and *E. coli* is probably ascribable to ferroptosis-like death, which provides the possibility for the development of antibacterial products.

### 2.4. Preparation and Characteristics of CSFe Hydrogel

**Figure 4:**
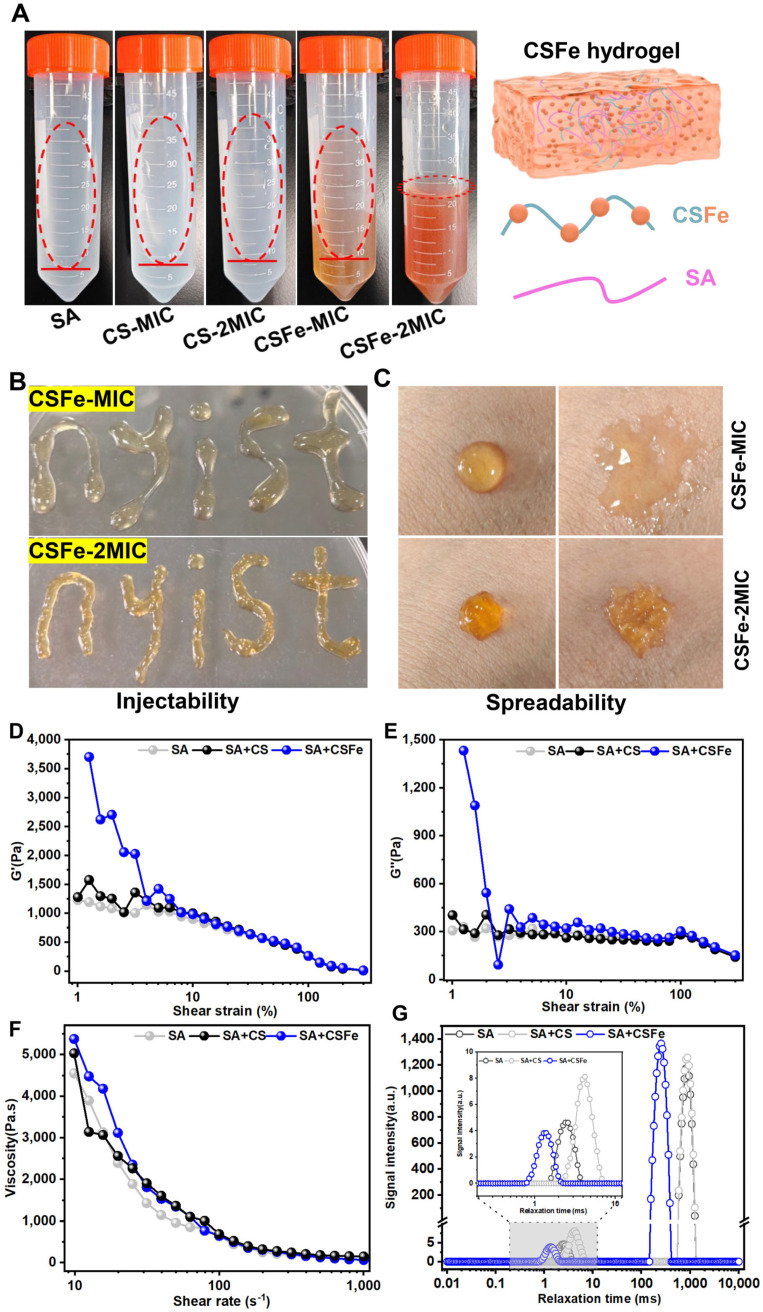
Fabrication and characteristics of CSFe hydrogel. (**A**) The status of SA, CS and CSFe hydrogels when tilted at about a 20−degree angle; (**B**) the injectability of CSFe hydrogel by syringe; (**C**) the spreadability of CSFe hydrogel on the surface of skin; rheological properties involved in the storage modulus (G′) (**D**), loss modulus (G″) (**E**) and viscosity (**F**) of the SA, CS and CSFe hydrogels at 2MIC; (**G**) T_2_ relaxation time distribution of the SA, CS and CSFe hydrogels at 2MIC. The dashed oval borders indicate the flow status of hydrogel samples.

It is difficult for CS to form hydrogel at a low concentration; this can be overcome by the use of crosslinking agents or other gel materials, such as SA [8]. Due to its anionic nature, SA has been extensively studied for hydrogel preparation. SA-based hydrogels are highly biocompatible and effective at absorbing wound exudate, and their gelation and crosslinking can be readily initiated through the exchange of Na^+^ with multivalent cations (e.g., Ca^2+^ or Fe^3+^) or via covalent bond formation [26]. In this study, a CSFe complex was incorporated into an SA matrix. Notably, Fe^3+^ ions present in the CSFe complex served as ionic crosslinkers that interact with the carboxylate groups of alginate chains, thereby promoting the formation of a stable three-dimensional gel network. CS hydrogels without CSFe were also prepared as controls. The appearance and properties of the resulting hydrogels are shown in Figure 4. Compared with hydrogels of pure SA and CS, CSFe hydrogel exhibits a greater viscosity, especially at a 2MIC concentration of CSFe, which presents with a jelly-like state (Figure 4A). Interestingly, the hydrogels of CSFe at MIC and 2MIC can be injected by syringe (Figure 4B) and spread on the surface of skin (Figure 4C). This means that CSFe hydrogel can be used on the surface of skin.

A rheological analysis of CSFe hydrogel at 2MIC, together with SA and CS as the control, suggests that the storage modulus (G′) values (Figure 4D) are much higher than the corresponding the loss modulus (G″) values (Figure 4E), and the values from the CSFe hydrogel are greater, which indicates that the CSFe hydrogel has a more relatively stable crosslinked structure. With the increase in shear rate, the viscosity of hydrogels of CSFe, CS and SA gradually decreases until becoming dynamically stable, suggesting these hydrogels have the excellent shear-thinning behavior. However, at a lower shear rate, the viscosity of CSFe hydrogels is significantly stronger than that of CS and SA hydrogels (Figure 4F). These results indicate that the CSFe hydrogel possesses a more stable crosslinked network and favorable shear-thinning behavior. The rheological characterization in this study did not include an amplitude sweep to verify the linear viscoelastic region (LVR) prior to modulus measurements. Therefore, it remains unclear whether the reported G′ and G′′ values were obtained entirely within the LVR. While the relative differences among the hydrogel formulations remain informative, future studies should incorporate an amplitude sweep analysis to ensure that all viscoelastic measurements are conducted under appropriate conditions.

The distribution and population of water within hydrogels can affect properties such as integrity, solubility, and substance diffusion. LF-NMR enables the assessment of different water types and their interactions with macromolecules in the hydrogel, where distinct relaxation times correspond to various modes of water molecule movement [8]. Generally, a shorter T_2_ relaxation time indicates lower water mobility and a higher degree of binding of the corresponding water molecules [27]. Here, only two peaks emerged in the range of 1–10,000 ms (Figure 4G). The peaks in the ranges of 0.1–10 ms, 15–500 ms, and >500 ms are recognized as bound water (T_21_), immobilized water (T_22_) and free water (T_23_). In terms of bound water (T_21_), the peak values of CSFe, CS and SA hydrogels are 1.289, 4.198 and 2.409 ms, respectively. The peak value corresponding to immobilized water (T_22_) is observed only in the CSFe hydrogel, at 252.353 ms. The peak values corresponding to free water (T_23_) are both 880.488 ms, and were observed in the CS and SA hydrogels. However, no peak (>500 ms) indicating free water (T_23_) was observed in the CSFe hydrogel. This means that the main water types in the CSFe hydrogel are bound water and immobilized water, suggesting that the CSFe hydrogel structure is more compact, probably due to the introduction of Fe^3+^ increasing the cross-linking of hydrogel.

### 2.5. Structure of CSFe Hydrogel

**Figure 5:**
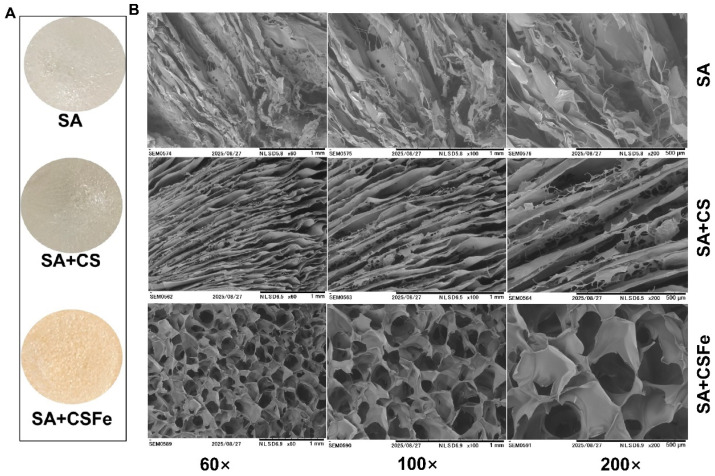
Morphology of CSFe hydrogel. The surface (**A**) and internal (**B**) structures of SA, CS and CSFe freeze-dried hydrogels.

Based on the results regarding the rheological properties and water distribution, it is speculated that the CSFe hydrogel possesses a more compact internal structure. In order to verify this judgment, the internal structures of the CSFe hydrogel, together with CS and SA hydrogels, were observed by SEM (Figure 5). Despite the difference in color, the surface morphologies of SA, CS and CSFe freeze-dried hydrogels are similar (Figure 5A). The internal structure of CSFe hydrogel exhibits a regular porous structure with a higher porosity and more compact three-dimensional network structure compared with that of the SA and CS hydrogels (Figure 5B). It was reported that in cartilage tissue engineering, the scaffolds should be highly porous with interconnected pore networks to facilitate cell attachment and proliferation [28]. Furthermore, scaffolds with higher porosity support essential biological processes such as cell migration, proliferation, gas exchange, as well as the transport of nutrients and removal of metabolic waste [29]. This means that the CSFe hydrogel has the potential to be used as a scaffold for tissue engineering. Additionally, the denser pore and compact structures of CSFe hydrogel probably result in an increase in the binding strength between water molecules and the polymer chains, and a restriction in the fluidity of the water immobilized inside the hydrogel, which could be the reason that the main water types in CSFe hydrogel are bound water and immobilized water, and the CSFe hydrogel has a higher viscosity.

### 2.6. Bactericidal Effect of CSFe Hydrogel

**Figure 6:**
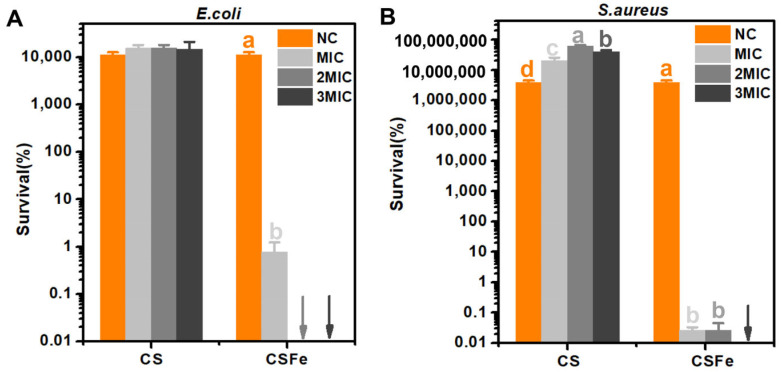
The bactericidal activity of CS and CSFe hydrogels against *E. coli* and *S. aureus*. The survival of *E. coli* (**A**) and *S. aureus* (**B**) exposed to CS and CSFe hydrogels (MIC, 2MIC, 3MIC) incubated in medium at 37 °C for 24 h. NC: The initial bacterial cell of *E. coli* and *S. aureus* without treatment of CS or CSFe, grown in medium under normal growth conditions for 24 h. When using the same treatment, the different letters indicate significant difference (*p* < 0.05). The arrows indicate no bacteria colony was observed on the plate.

The antibacterial ability of CSFe was confirmed by the results mentioned above. However, it is not clear whether the bactericidal activity, especially the bactericidal effect, of CSFe will be influenced after it is made into hydrogel. Therefore, the bactericidal effects of different concentrations of CSFe (MIC, 2MIC and 3MIC) in hydrogel on *E. coli* and *S. aureus* were investigated. In terms of *E. coli* (Figure 6A), the CS hydrogel hardly affects normal growth, and *E. coli* can proliferate at the normal growth conditions (when containing LB medium). However, the CFU of *E. coli* treated with CSFe hydrogel cannot be observed at the concentrations of the CSFe of 2MIC and 3MIC in hydrogels. Similarly, CS hydrogel fails to affect the growth of *S. aureus*, and cells of *S. aureus* can proliferate with normal growth conditions (when containing TSB medium) (Figure 6B). the CFU of *S. aureus* treated with hydrogel cannot be observed at the 3MIC concentrations of CSFe in hydrogel. The bactericidal rates of CSFe hydrogel against *S. aureus* reach 99.97% at MIC and 2MIC. Interestingly, based on the presented results, the bactericidal activity of CSFe hydrogel seems to be stronger than that of CSFe. Overall, CSFe made into hydrogel does not decrease bactericidal activity against *E. coli* and *S. aureus*. This means that this CSFe hydrogel can be employed as an antibacterial material.

### 2.7. CSFe Hydrogel’s Ability to Promoted Infected-Wound Healing

**Figure 7:**
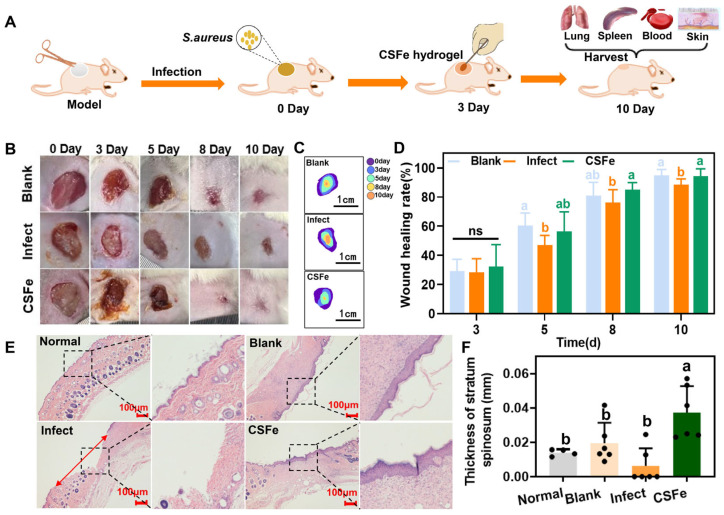
The infected-wound healing effects of CSFe hydrogel. (**A**) Schematic diagram of the details of *S. aureus*-infected wound test in vivo; (**B**) digital wound photographs of different groups on days 0, 3, 5, 8 and 10; (**C**) wound healing areas in different groups, obtained by ImageJ (ImageJ 1.53a, Wayne Rashband National Institute of Health, USA); (**D**) wound healing rate in different groups at 3, 5, 8 and 10 days; (**E**) H&E staining of the wound skin; (**F**) thicknesses of the stratum spinosum. The different letters indicate significant difference among the different groups (*n* = 6 per group, *p* < 0.05), and “ns” indicates no significance. The red arrow represents unhealed wound tissue.

CSFe hydrogel’s ability to promote infected-wound healing was assessed on the full-thickness skin wound model infected by *S. aureus*. The wounds were infected by *S. aureus* (infected group) or not (blank group), and the infected wound was treated with CSFe hydrogel as the CSFe group. The blank group was used to simulate the natural wound healing process. The whole simplified experimental process is shown in Figure 7A. The wound pictures and quantification of wound healing rates on days 0, 3, 5, 8 and 10 indicate that CSFe hydrogel dramatically promotes the infected-wound healing in mice (Figure 7B–D). The healing rate of the CSFe hydrogel group is 86.8%, which is significantly (*p* < 0.05) higher than that of the infected group (77.8%) on day 8. On day 10, the wound healing rate of the CSFe hydrogel group is 95.8%, which is still higher (*p* < 0.05) than the infected group (90.2%). The H&E staining results of the wound skin show that the wound morphologies in the blank, infected and CSFe groups are different from the normal skin tissue (Figure 7E). Specifically, the healed wound in the blank and CSFe groups can be observed while the wound failed to heal in the infected group. Interestingly, the skin stratum spinosum of the CSFe hydrogel group is significantly thicker than that of other groups (Figure 7F). Additionally, statistical analysis was conducted on the viscera index including the lung and spleen (Figure S3A,B). No significant difference in the spleen index and lung index is observed among blank, infected and CSFe groups. Taken together, CSFe hydrogel treatment is able to promote infected-wound healing, achieving a natural wound healing ability.

**Figure 8:**
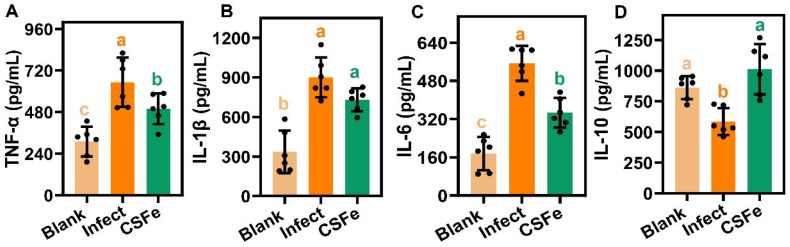
The changes in inflammatory factors in the serum of the mice among blank, infected and CSFe groups. The levels of pro-inflammatory factors, including TNF-α (**A**), IL-1β (**B**) and IL-6 (**C**), in serum of the different groups; (**D**) the levels of the anti-inflammatory factor of IL-10 in the serum of the different groups. The different letters indicate significant difference among the different groups (*n* = 6 per group *p* < 0.05).

To further evaluate the anti-infection effects of CSFe hydrogel, the inflammatory factors of TNF-α, IL-1β, IL-6 and IL-10 were detected on day 10. The results are shown in Figure 8. The pro-inflammatory factors of TNF-α, IL-1β and IL-6 in the infected group are significantly (*p* < 0.05) increased compared with the blank group while CSFe hydrogel treatment decreases (*p* < 0.05) the levels of TNF-α and IL-6 (Figure 8A–C). On the contrary, the anti-inflammatory factor of IL-10 in the CSFe hydrogel group is significantly (*p* < 0.05) increased compared with the infected group (Figure 8D).

*S. aureus*, a kind of human pathogen, is the most commonly found bacteria in wound contamination, largely owing to its remarkable adaptability and ability to acquire antimicrobial resistance genes [1]. The results regarding the antibacterial ability of CSFe and its hydrogel indicate that CSFe hydrogel material has the potential to improve infected-wound healing. Here, using a mouse model of *S. aureus*-infected wounds, we confirmed that the CSFe hydrogel promoted the healing of skin wounds. Recent studies suggested that ferrous sulfate directly loaded in hydrogel exhibited good antibacterial ability and facilitated wound healing; this was ascribed to the ferroptosis-like cell death induced by ferrous sulfate [24,30]. In fact, Fe^3+^-doped nanozymes could also relieve inflammation and *S. aureus* infection levels in mice [23]. It was suggested that both Fe^3+^ and Fe^2+^ show antibacterial ability against *S. aureus*. *S. aureus*-infected wounds cause a significant inflammatory response, and therefore, the anti-inflammatory effects of CSFe hydrogel on *S. aureus* was also investigated. *S. aureus* triggers inflammation via the activation of recognition receptors, resulting in the upregulation of pro-inflammatory factors like TNF-α, IL-1β, and IL-6 [1]. It was recognized that these pro-inflammatory cytokines were secreted by M1 macrophages from the wound, and the anti-inflammatory cytokine of IL-10 was due to M2 macrophages [4]. In the present study, CSFe hydrogel treatment could decrease the pro-inflammatory properties of TNF-α and IL-6, and increase the anti-inflammatory properties of IL-10 in serum. These results suggest that the changes in cytokine levels are associated with the accelerated wound healing process following CSFe hydrogel treatment. While a direct anti-inflammatory effect of the locally applied hydrogel cannot be concluded, the observed modulation of inflammatory cytokines indicates a favorable immunomodulatory response accompanying enhanced tissue repair.

## 3. Conclusions

Fe^3+^ can interact with CS to form a structurally stable polysaccharide–metal complex (CSFe), which exhibits an iron-holding capacity of 2.06%. Due to its antibacterial activity, CSFe is suitable for applications in the antibacterial field. A CSFe hydrogel with antibacterial properties was fabricated by combining CSFe with SA. This hydrogel possesses a porous three-dimensional structure and can promote infected-wound healing involving the modulation of inflammation. However, the limit of this study is the lack of an antibacterial mechanism of CSFe against *E. coli* and *S. aureus*, which should be overcome in future research. While the present study demonstrates the potent antibacterial activity of CSFe against *E. coli* and *S. aureus*, further investigation using a broader panel of bacterial strains, including clinically relevant multidrug-resistant pathogens, would be valuable to fully characterize its antibacterial spectrum. We also acknowledge the absence of a positive control group in the animal experiment, as well as the qualitative nature of the injectability and spreadability assessments, and the lack of an amplitude sweep verification for the linear viscoelastic region in rheological characterization. These limitations will be addressed in future studies to provide a more comprehensive assessment. This study provides insights for evaluating the activity and elucidating the mechanisms of polysaccharide–metal complexes. It also contributes to the development of novel antibacterial materials to address microbial resistance, supporting the discovery of innovative biomedical materials.

## 4. Materials and Methods

### 4.1. Materials

In this study, two polysaccharides were used: CS and SA. CS was sourced from bovine cartilage and was provided by the Institute of Food Science and Technology, Chinese Academy of Agricultural Sciences. Its key parameters, including uronic acid content (determined by the carbazole–sulfuric acid method), sulfate group content (determined by ion chromatography), and disaccharide composition (analyzed by strong anion-exchange high-performance liquid chromatography after enzymatic digestion), are presented in Table S1. SA (S817374) was purchased from Shanghai Macklin Biochemical Co., Ltd., (Shanghai, China). Its characterization data are also presented in Table S2. The iron chloride hexahydrate (FeCl_3_·6H_2_O, purity: 99%) used was supplied by Tianjin Kemiou Chemical Reagent Co., Ltd., Tianjin, China. *S. aureus* (ATCC 25923) and *E. coli* (MG1655) were provided by Shaanxi University of Science and Technology. All the other chemicals were analytical grade.

### 4.2. Preparation of CSFe

CSFe was prepared based on previous studies with some modifications, which mainly included ion exchange (such as Na^+^ and Fe^3+^), the reaction of CS and FeCl_3_, and the precipitation of CSFe [11]. The flow chart is shown in Figure 1A. Specifically, 20 g CS was completely dissolved in 800 mL deionized water to prepare 2.5% CS solution, and then FeCl_3_·6H_2_O [CS (g): FeCl_3_·6H_2_O (mmol) = 1:0.25] was added to 800 mL 2.5% CS solution for 24 h. CSFe was precipitated from the liquid sample by adding three volumes of ethanol, with repeated operations until the supernatant became clear to eliminate residual FeCl_3_. After being redissolved in deionized water, the sample was dialyzed (10 kDa) for 96 h to remove unbound ferric ions, and finally lyophilized to obtain CSFe powder.

### 4.3. Characterization of CSFe

The morphology of CSFe and CS powder samples was examined via scanning electron microscopy (SEM, COXEM-30 plus, Daejeon, Republic of Korea). For sample preparation, a small amount of each powder was fixed onto aluminum stubs and coated with gold to enhance conductivity, followed by SEM observation at 9 kV using magnifications of 500×, 1000×, 2000×, 5000×, and 12,000×. Elemental composition was determined by energy-dispersive spectroscopy (EDS, OXFORD IE250, Oxford, UK) integrated with SEM. X-ray diffraction (XRD, D8 Advance ECO, Bruker, Karlsruhe, Germany) was used to analyze crystal structures, with X-rays generated at 30 kV and 10 mA, and patterns recorded in the 2θ range of 10–90°.

A molecular weight (Mw) analysis of CSFe and CS was conducted via gel permeation chromatography coupled with multi-angle laser light scattering. The system featured an Agilent 1260 Infinity II HPLC (Agilent Technologies, Inc., Waldbronn, Germany), a BI-MwA multi-angle light scattering detector, a differential detector, and a PL aquagel–OH column (300 × 7.5 mm). All temperature-controlled components—including the column oven, light scattering detector, and refractive index detector—were set to 30 °C. Separation was performed using 0.1 M sodium chloride as the mobile phase at 0.5 mL/min. Samples were dissolved in the mobile phase at 1 mg/mL, and 10 µL was injected for analysis. Detector calibration was achieved using a dextran standard (Mw = 102,000 g/mol) prepared in the mobile phase.

### 4.4. Fe Holding Capacity and Zeta Potential of CSFe

The iron content of CSFe and CS samples was determined by inductively coupled plasma mass spectrometry (ICP-MS, 7700×, Agilent Technologies Inc., Palo Alto, CA, USA) in conjunction with a microwave digestion system (MARS 5, CEM Corp., Matthews, NC, USA) and an auto-sampler, allowing for assessment of the iron-binding capacity of CSFe. The Zeta potential of CSFe and CS was estimated at a concentration of 2.5 mg/mL by a Zeta potential analyzer (Nano9200, HAIXINRUI, Beijing, China) at room temperature.

### 4.5. FTIR, 1H-NMR and Thermal Properties of CSFe

Characterization using FTIR was conducted on a Nicolet Summit X spectrometer (Thermo Fisher Scientific, Madison, WI, USA), where dried CSFe or CS samples were mixed with KBr (1:200), pressed into ~1 mm pellets, and scanned in transmission mode from 4000 to 400 cm^−1^ at a resolution of 4 cm^−1^. ^1^H-NMR analysis was carried out using a Bruker AMX 400 MHz spectrometer with a 5 mm tunable probe, and samples were prepared by dissolving 50 mg of CSFe or CS in 1 mL of D_2_O. The spectra of CSFe and CS were compared and analyzed with MestReNova software. About 20 mg of the CSFe or CS samples was taken to determine the thermal properties using a simultaneous thermal analyzer (DZ-STA200, Nanjing, China) from 25 to 600 °C at a rate of 20 °C/min under N_2_ atmosphere.

### 4.6. Determination of Antibacterial Activity of CSFe

*E. coli* was cultured in Luria–Bertani (LB) medium, and *S. aureus* was cultured in Trypticase Soy Broth (TSB) medium. The two strains were cultured at 37 °C, and the cell growth was evaluated using an optical density of 600 nm (OD600) via spectrophotometer. The effects of CSFe and CS on the growth of *E. coli* and *S. aureus* were determined by the broth microdilution method with modifications [31]. Specifically, cells (approximately 10^6^ CFU/mL) of *E. coli* or *S. aureus* at exponential phase were added to each well of a 96-well plate, then exposed to a series of concentrations at 0, 2, 4, 6, 8, 10, 12 mg/mL and incubated at 37 °C for 72 h. Each well of OD600 was recorded using a microreader to evaluate the minimum inhibitory concentrations (MIC) of CSFe on *E. coli* or *S. aureus*. The MIC of CSFe on *E. coli* or *S. aureus* was defined as the lowest concentration that inhibited visible growth.

The drop plate method was employed to assess the bactericidal activity of CSFe against *E. coli* or *S. aureus*. Briefly, cells (approximately 10^6^ CFU/mL) of *E. coli* or *S. aureus* containing the normal medium were treated with 0MIC (NC), 1MIC, 2MIC and 3MIC of CSFe, together with the same concentrations of CS as the control, and incubated at 37 °C for 6 h and 12 h. After being cultured, eight dilutions of each treatment were plated on LB or TSB agar incubated at 37 °C for about 12 h. The survival ratio was calculated as Equation (1):

(1) $$\mathrm{Survival}\;\mathrm{ratio}\;\left(\%\right)=\frac{{CFU}_{t}}{{CFU}_{0}}\;\times 100$$

where CFU*_t_* indicates the number of the colony-forming units of *E. coli* or *S. aureus* treated with CSFe or CS, and CFU_0_ indicates the initial number of bacterial cells (approximately 10^6^ CFU).

### 4.7. Preparation of CSFe Hydrogel

The CSFe hydrogel was prepared based on the method of Wang et al., with some modifications [24]. Briefly, different concentrations of CSFe solution (0, MIC, 2MIC) were prepared, and then 1% (*w*/*v*) sodium alginate (SA) was added with vigorous shaking until a clear solution was obtained. In this system, Fe^3+^ ions derived from the CSFe complex acted as ionic crosslinkers, interacting with the carboxylate groups of alginate chains to form a three-dimensional gel network. The hydrogel was then subjected to vacuum degassing to remove bubbles and stored at 4 °C until further use. CS hydrogels at the same concentrations were also prepared as controls.

### 4.8. Characterization of CSFe Hydrogel

The mobility of CSFe and CS hydrogel was evaluated by the 20 mL hydrogel system prepared using the above method. A total of 20 mL of different concentrations (0, MIC, 2MIC) of CSFe and CS hydrogel in a 50 mL centrifuge tube were tilted at a 20-degree angle to observe their mobility. The injectability of CSFe hydrogel was assessed by loading 1 mL of CSFe hydrogel (MIC and 2MIC) into a syringe followed by injecting it into a plastic dish. The spreadability of CSFe hydrogel was estimated by dropping and smearing on human skin.

The morphologies of CSFe hydrogel together with CS hydrogel were observed by Scanning Electron Microscopy (SEM) (JSM-7610FPlus, JEOL Ltd. Tokyo, Japan). Before SEM, CSFe hydrogel together with CS hydrogel was freeze-dried and a thin cross-section was cut out. The thin cross-section of hydrogel was fixed on the aluminum sample stubs and coated with gold to enhance conductivity for SEM. The morphology of hydrogel was observed at 2 kV extra-high tension with various magnifications of 60×, 100×, 200×.

The rheological properties of CSFe hydrogel together with CS hydrogel were analyzed by rheometer (RH20, BosinTech, Shanghai, China) equipped with a CP50 cone–plate rotor (50 mm diameter, 1° cone angle) at a constant temperature of 25 °C with constant-temperature circulating water. The gap between the rotor and the platform was set to 1 mm. For modulus measurements, the oscillation frequency was fixed at 10 Hz, and the shear strain was varied from 1% to 300%. The viscosity was determined with the shear rate of 1–1000 s^−1^.

Low-field nuclear magnetic resonance (NMI20-060H-I, Suzhou Niumag Co., Suzhou, China) was used to measure water mobility in the CSFe hydrogel, which was placed in a nuclear magnetic resonance tube. The instrument was operated with a 0.5 T magnetic field at a resonance frequency of 200 MHz, and transverse relaxation curves (*T*_2_) were acquired using the Carr–Purcell–Meiboom–Gill sequence.

### 4.9. Bactericidal Activity of CSFe Hydrogel

The bactericidal activity of CSFe hydrogel was determined by a 1 mL hydrogel system containing SA (1%) and cells (approximately 10^6^ CFU/mL) of *E. coli* or *S. aureus*, with or without CSFe (MIC, 2MIC, 3MIC), and the same concentrations of CS as the control. The hydrogel system was incubated at 37 °C, 200 rpm, for 24 h. After incubation, 1 mL sterile saline was added to the hydrogel and mixed thoroughly to count the CFU of *E. coli* or *S. aureus* when exposed to various concentrations of CSFe hydrogel by the drop plate method described above.

### 4.10. Animal Experiment Design

This study utilized five-week-old male SPF KM mice purchased from Henan Skobes Biotechnology Co., Ltd. (Anyang, China). Animals were housed in an SPF-grade facility under a 12 h light/dark cycle with 50–70% relative humidity, and had ad libitum access to standard diet and water. All procedures were approved by the Ethics Committee of Nanyang Institute of Technology (No.: 2025002) and conformed to the European Community guidelines (2010/63/EU) for laboratory animal care.

Prior to the experiment, the mice underwent a one-week acclimatization period to become accustomed to the housing conditions, with three animals per cage. Subsequently, they were randomly divided into three experimental groups (*n* = 18), including the blank group (*n* = 6), who only suffered from wound surgery, the infected group (*n* = 6), who suffered from wound surgery and were infected with *S. aureus*, and the CSFe group (*n* = 6), who suffered from wound surgery, were infected by *S. aureus*, and were treated with CSFe hydrogel. The wound surgery operation was based on the method of Wang et al., with some modifications [24]. Specifically, all the mice were anesthetized via isoflurane inhalation, and the dorsal hair was carefully shaved and disinfected with 75% ethanol. A full-thickness skin wound with a diameter of 10 mm was created in the center of the dorsal flank using a sterile biopsy punch. The wound was excised through the full thickness of the skin down to the loose subcutaneous tissue, while preserving the underlying muscle layer. To ensure the standardization of wound size and depth, all surgical procedures were performed by the same operator using identical instruments, and the wound dimensions were immediately measured after creation to confirm consistency across all animals. Then, the mice in the infected and CSFe groups were infected by *S. aureus* (approximately 10^10^ CFU/mL) for three days. Specifically, after wound creation, 100 μL of *S. aureus* suspension was pipetted onto the wound surface and allowed to be naturally absorbed. The wound was then covered with sterile gauze to maintain moisture and promote bacterial colonization. This procedure was repeated daily for three consecutive days. The successful establishment of the infected wound model was confirmed by observing the presence of suppuration around the wound margin. After the infection phase, the CSFe group mice were treated with CSFe hydrogel (once a day) for 10 days. Approximately 200 μL of CSFe hydrogel was applied evenly to cover the entire wound surface using a sterile cotton stick. The wound was then left uncovered to allow for direct observation and to avoid any confounding effects from bandage materials. For consistency, all groups (blank, infected, and CSFe) were treated under the same conditions without bandaging. The wound was measured and imaged every day. Finally, the skin tissue around the wound, blood and organs, including the lung and spleen, were taken for the following assays.

### 4.11. Animal Sample Collection and Determination

After the experiment, the bodyweight of each mouse was recorded, and the mice suffered from a 12 h fasting period before the collection of their blood and organs. Firstly, mice were anesthetized with isoflurane. Blood samples were obtained from the orbital vein, and serum was separated by centrifugation at 2000 *g* for 10 min. The serum was also stored at −80 °C until ready for the determination of inflammatory factors. The inflammatory factors of tumor necrosis factor-α (TNF-α, MU30030), interleukin-1β (IL-1β, MU30369), interleukin-6 (IL-6, MU30044) and interleukin-10 (IL-10, MU30055) were determined by mouse ELISA kits (Bioswamp^®^, Bioswamp Life Science Lab, Wuhan, China) based on the manufacturer’s instructions. The lung and spleen were taken and weighed. The skin tissue around the wound was taken, and then fixed in 10% formalin, processed into paraffin-embedded wax blocks, sectioned, and stained with hematoxylin and eosin (H&E). The viscera index was calculated by Equation (2):

(2) $$\mathrm{Viscera}\;\mathrm{index}\;\left(\%\right)=\frac{\mathrm{Weight}\;\mathrm{of}\;\mathrm{organ}\;(g)}{\mathrm{Bodyweight}\;(g)}\;\times \;100$$

### 4.12. Statistical Analysis

Statistical analysis was carried out with SPSS 2.0 software. One-way ANOVA followed by Duncan’s multiple range tests was used for multiple-group comparisons, whereas Student’s *t*-test was employed for two-group comparisons. Data are shown as mean ± standard deviation (SD). Each group in the in vivo wound healing assay consisted of six mice (*n* = 6). Statistical significance was defined as * *p* < 0.05 and ** *p* < 0.01.

## Figures and Tables

**Table 1:** Some published articles showing hydrogels in the composition, the target and the main results.

Main Composition	Target	Main Results	Reference
CS/hydrazide	To prevent postoperative adhesions	Significantly reduced adhesions in rat models	[7]
CS or oxidized chondroitin sulfate (CSO)	To investigate how CS incorporation affects mechanical properties and fibrochondrogenic differentiation for tendon-to-bone enthesis repair	Both CS and CSO increased hydrogel compliance; free CS promoted pro-enthesis gene expression and altered local strain distribution in triphasic scaffolds; CSO largely suppressed enthesis-related gene expression	[13]
carboxymethyl chitosan/sodium alginate/mesenchymal stem cell exosomes	To promote diabetic wound healing by scavenging excess ROS, enabling ROS-responsive exosome release, and modulating macrophage polarization from M1 to M2 phenotype.	Hydrogel showed rapid gelation, injectability, self-healing, tissue adhesion, and ROS-responsive degradation; hydrogel @Exos enhanced cell migration, reduced inflammation, promoted M2 macrophage polarization, and significantly accelerated wound closure in diabetic mice	[14]
Sodium alginate/hyaluronic acid/gelatin/carboxymethyl chitosan/antimicrobial peptide	To develop a multifunctional wound dressing that integrates antibacterial, anti-inflammatory, and pro-healing properties to enhance wound repair	Hydrogel exhibited good injectability, degradability, sustained release, broad-spectrum antibacterial activity, and low hemolysis, and promoted cell migration, angiogenesis, and wound closure in vivo with no organ toxicity	[15]

## Data Availability

The original contributions presented in this study are included in the article and Supplementary Material. Further inquiries can be directed to the corresponding author.

## Supplementary Materials

The following supporting information can be downloaded at: https://www.mdpi.com/article/10.3390/gels12040329/s1, Figure S1: The bactericidal activity of CS and CSFe to *E. coli*; Figure S2: The bactericidal activity of CS and CSFe to *S. aureus*; Figure S3: The viscera index of lung and spleen; Table S1: The information of CS from bovine cartilage [32]; Table S2: Characterization data of SA (S817374).
