# Supplementary material for: A Chondroitin Sulfate–Iron Complex with Antibacterial Activity and Its Derived Hydrogel for Infected Wound Healing
Source: Gels. 2026 Apr 15;12(4):329. doi: 10.3390/gels12040329 (PMC13116974; doi:10.3390/gels12040329)
Supplement: Supplementary file 1 [file gels-12-00329-s001.zip › gels-4240579-supplementary.pdf]

A Chondroitin Sulfate–Iron Complex with Antibacterial Activity and Its Derived Hydrogel for Infected Wound Healing

Qingshan Shen[1], Yujie Dong[1], Jiawen Li[1], Jiarui Wu[1], Chengzhi Hu[2], Yang Liu[1], Lei Zhao[1], Huan Zhan[1], Hua Bian[1], Yanli Ma[1, *]

[1]Henan Key Laboratory of Zhang Zhongjing Formulae and Herbs for Immunoregulation, Zhang Zhongjing College of Chinese Medicine, Nanyang Institute of Technology, Changjiang Road 80, Nanyang 473004, China.

[2]College of Food Science and Technology, Hebei Agricultural University, Baoding 071000, China.

Table S1 The information of CS from bovine cartilage [32].

Category	Contents (%)						
Uronic acid	31.87 ± 1.27						
Sulfate	18.39 ± 0.10						
Molar ratio	GalNAc	GlcA	ΔDi0S	ΔDi6S	ΔDi4S	ΔDi2, 6S	4S/6S
	33.18	55.51	3.02	32.24	64.74	--	2.01

Note: GlcA, D-glucuronic acid; GalNAc, N-acetyl-D-galactosamine; ΔDi, unsaturated disaccharide unit; S, sulfate group.

Table S2 Characterization data of SA (S817374)

	Min.	Max.
Purity (%)	90	100
Lead (Pb) (ppm)	0	20
Loss on Drying (%)	0	15
PH (1%,20°C)	5	8
Sulfated Ash (%)	20	36.0
Appearance	White to brown or yellow powder	

Reference

32. Wang, K.; Wang, W.; Zhang, R.; Liu, Y.; Hou, C.; Guo, Y.; Zhang, C. Preparation of low molecular weight chondroitin sulfate from different sources by H_2O_2/ascorbic acid degradation and its degradation mechanism. *Food Chem,* **2024**, *434*, 137392.

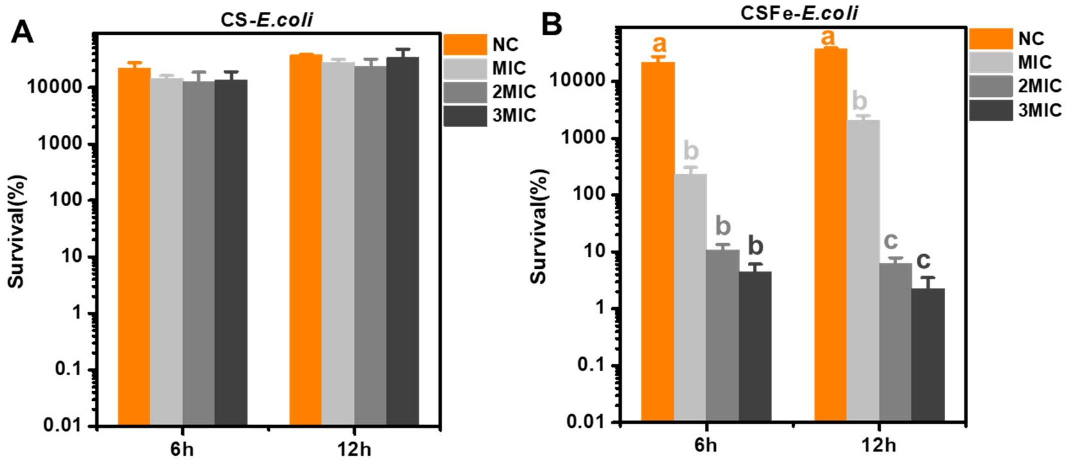

Figure S1. The bactericidal activity of CS and CSFe to *E. coli*. The survival of *E. coli* exposed to CS (A) and CSFe (B) (MIC, 2MIC, 3MIC) incubated at 37 °C for 6 h and 12h. NC: The initial bacterial cells of *E. coli* and *S. aureus,* without treatment with CS or CSFe, grown in medium under normal growth conditions for 6 h or 24 h. At the same time point, different letters indicate significant difference (P<0.05).

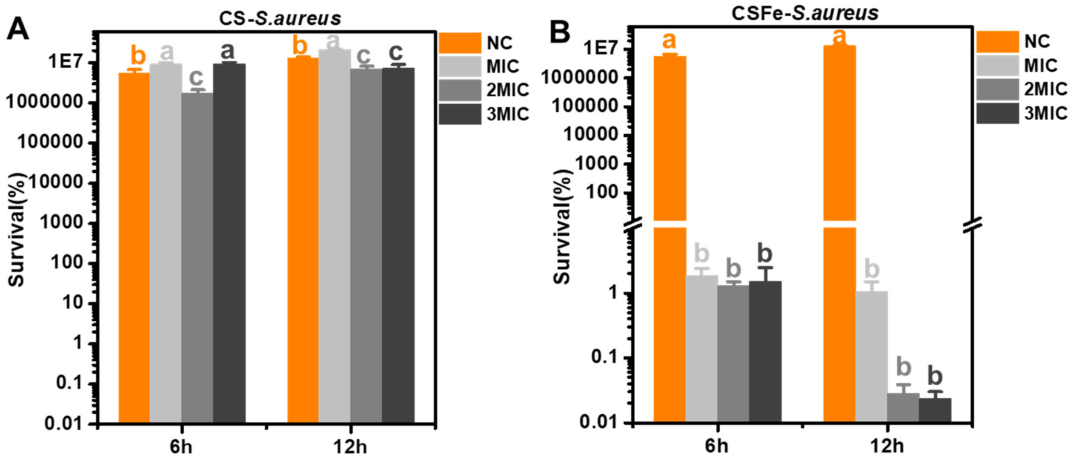

Figure S2. The bactericidal activity of CS and CSFe to *S. aureus*. The survival of *S. aureus* exposed to CS (A) and CSFe (B) (0, MIC, 2MIC, 3MIC) incubated at 37 ℃ for 6 h and 12h. NC: The initial bacterial cells of *E. coli* and *S. aureus* without treatment with CS or CSFe, grown in medium under normal growth conditions for 6 h or 24 h. At the same time point, different letters indicate significant difference (P<0.05).

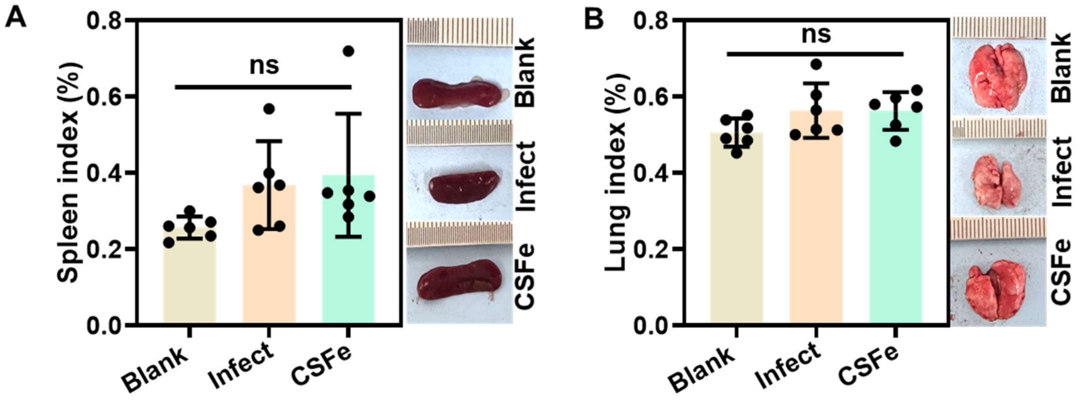

Figure S3. The viscera index of lung and spleen. The spleen index (A) and lung index (B) of the blank, infected and CSFe groups. ns indicates no significant difference among the three groups (P>0.05).
